# Multi-class computational evolution: development, benchmark evaluation and application to RNA-Seq biomarker discovery

**DOI:** 10.1186/s13040-017-0134-8

**Published:** 2017-04-24

**Authors:** Nathaniel M. Crabtree, Jason H. Moore, John F. Bowyer, Nysia I. George

**Affiliations:** 1 Bioinformatics, Department of Information Science, University of Arkansas at Little Rock and University of Arkansas for Medical Sciences Joint Bioinformatics Graduate Program, Little Rock, AR USA; 2 Division of Informatics, Department of Biostatistics and Epidemiology, Institute for Biomedical Informatics, Perelman School of Medicine, University of Pennsylvania, Philadelphia, PA 19104-6021 USA; 3 Division of Neurotoxicology, National Center for Toxicological Research, FDA, Jefferson, AR USA; 4 Division of Bioinformatics and Biostatistics, National Center for Toxicological Research, FDA, Jefferson, AR USA

**Keywords:** Artificial intelligence, Feature selection, Classification, Genetic programming, Machine learning, Data mining, Biomarker discovery, Evolutionary algorithm, Multi-class

## Abstract

**Background:**

A computational evolution system (CES) is a knowledge discovery engine that can identify subtle, synergistic relationships in large datasets. Pareto optimization allows CESs to balance accuracy with model complexity when evolving classifiers. Using Pareto optimization, a CES is able to identify a very small number of features while maintaining high classification accuracy. A CES can be designed for various types of data, and the user can exploit expert knowledge about the classification problem in order to improve discrimination between classes. These characteristics give CES an advantage over other classification and feature selection algorithms, particularly when the goal is to identify a small number of highly relevant, non-redundant biomarkers. Previously, CESs have been developed only for binary class datasets. In this study, we developed a multi-class CES.

**Results:**

The multi-class CES was compared to three common feature selection and classification algorithms: support vector machine (SVM), random k-nearest neighbor (RKNN), and random forest (RF). The algorithms were evaluated on three distinct multi-class RNA sequencing datasets. The comparison criteria were run-time, classification accuracy, number of selected features, and stability of selected feature set (as measured by the Tanimoto distance). The performance of each algorithm was data-dependent. CES performed best on the dataset with the smallest sample size, indicating that CES has a unique advantage since the accuracy of most classification methods suffer when sample size is small.

**Conclusion:**

The multi-class extension of CES increases the appeal of its application to complex, multi-class datasets in order to identify important biomarkers and features.

**Electronic supplementary material:**

The online version of this article (doi:10.1186/s13040-017-0134-8) contains supplementary material, which is available to authorized users.

## Background

In this work, an existing computational evolution system (CES) for binary classification [1] was extended to accommodate multi-class problems. Although several multi-class classification algorithms exist, the CES has advantages in that it performs better on small-sample datasets and requires fewer features to do so. As well, the selected features may be more specific and thus better biomarkers for treatment response or disease diagnosis.

The goal of supervised classification is to build a model that can accurately predict the class membership of a new observation based on a training dataset where the class labels are known. Common classification algorithms can be broadly categorized as decision trees, nearest neighbor methods, linear classifiers (e.g. linear discriminant analysis and naïve Bayes classifier), and support vector machines. When classifying high-dimensional data (i.e. ‘large p, small n’ settings), better performance and interpretability is achieved through feature selection, which is a dimensionality reduction technique by which a small, relevant subset of the original features is selected based on certain evaluation criterion. Feature selection techniques such as filter methods are performed as a data preprocessing step and implemented independent of classifier learning. Filter methods do not consider feature interaction, which will likely result in suboptimal classifiers. Alternatively, many classification algorithms identify a set of discriminative features by performing both feature selection and model fitting (e.g. wrapper and hybrid methods), which typically leads to better accuracy and efficiency.

There are two general approaches to multi-class classification algorithms. The first approach is to decompose the multi-class setting into several binary problems, as is typically done for multi-class support vector machines (SVM) [2, 3]. Binary datasets may be constructed by either pairing one class against the rest (i.e. one-versus-rest, OVR) or by pairing one class against another class and considering all possible pairwise binary problems (i.e. one-versus-one, OVO). In the latter strategy, class prediction is determined through majority voting; whereas, in the former, class prediction is determined by highest probability. OVR may not be appropriate for certain algorithms because it can create an imbalanced class distribution. Furthermore, even if class size is balanced by random sampling from the larger class, the ‘rest’ class will be comprised of multiple different classes, which may make it difficult for the classifier to perform well [4]. On the other hand, the OVO strategy is regarded for its computational efficiency. Alternatively, the second approach to multi-class classification is to process all the data/classes at once, allowing for a natural extension between binary and multi-class classification. For example, methods such as K-nearest neighbor (KNN) and decision trees extend naturally from the binary to multi-class setting.

In this regard, the multi-class CES was developed to discriminate between multiple classes without using a decomposition approach. The creation of CES started with the development of symbolic discriminant analysis (SDA), a modification of Fisher linear discriminant analysis (FLDA). SDA borrowed from FLDA the idea of using a discriminant function and threshold value to predict the class membership of samples [5]. FLDA was limited to linear functions, which cannot accurately model nonlinear relationships among variables, while SDA allowed nonlinear discriminant functions taking any form, required no pre-specification of a model, and made no assumptions about the data [6]. The SDA method was further enhanced with genetic programming to allow coarse-grained searches of the problem space, saving run-time. Genetic programming algorithms use basic building blocks (e.g. functions, constants, mathematical operators, and features) to construct new features and evaluate them according to a fitness function. Mutation functions modify classifiers by adding or removing building blocks or by swapping building blocks in different ways and at varying frequencies according to the specified parameters [7]. This flexibility in the model building process is controlled by a meta-layer that learns how to adjust parameters to build better models in a way similar to how a human would tinker with the data given infinite time [1]. Classifiers that perform best according to the fitness function are further evolved by adding or removing building blocks or by combining multiple classifiers into a single, new classifier. The process is repeated through many rounds of evolution until a best classifier or best set of classifiers is produced [8]. It should be noted that the fitness function utilized by CES considers the balance between classification accuracy and model complexity, which is measured by the number of building blocks.

Finally, Pareto optimization and post-processing were integrated with SDA by Moore et al. [1] to identify optimal solutions for multi-criterion optimization and prevent over-fitting. Pareto optimization identifies the Pareto front [1], which is the subset of solutions with highest classification accuracy and lowest model complexity. Post-processing steps were implemented to allow the results of one CES run to be used as input to subsequent CES runs. Pareto domination tournament as described by Horn [9] was utilized to generate solutions. Briefly, the solutions are generated on a grid, where each solution has eight immediate neighbor solutions in its neighborhood. Solutions that dominate the neighborhood repopulate all eight neighboring grid locations in the next round of evolution, after being mutated. A solution is considered dominant based on the fitness function.

Given the advantages of the CES, in this work, we have developed a multi-class CES and present a comparative analysis of CES with three competing feature selection and classification algorithms for multi-class data: SVM, random KNN (RKNN), and random forest (RF). The methods are evaluated using three distinct multi-class RNA sequencing (RNA-Seq) datasets and are assessed in terms of classification accuracy, number of selected features, and stability of the selected features.

## Methods

### Generalizing the binary CES algorithm to multiple classes

**Fig. 1:**
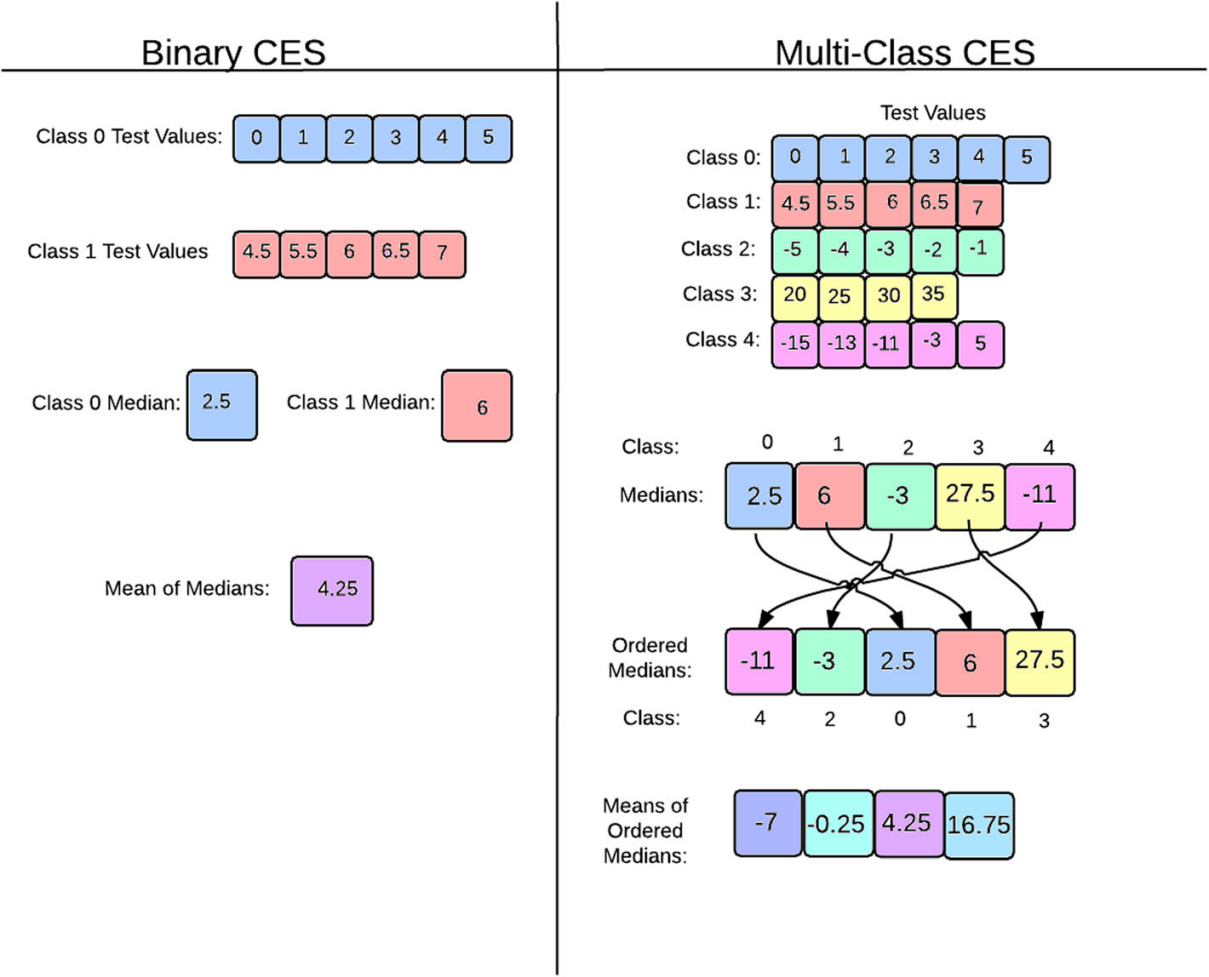
Differences between the binary and multi-class CES classification algorithm. Description of data: The binary-class CES classification algorithm was generalized to the multi-class problem using flexible data structures and by sorting each class according to the mean of the median test value. Example test values are provided

In order to accomplish multi-class, all-at-once classification, the software containers and classification algorithms were generalized to hold and process multi-class data. Specifically, the array data structure used in the binary problem was generalized to a two-dimensional array of arrays that can grow or shrink to hold the test values for a variable number of classes. In addition, the classification rule for the multi-class problem was determined using the median test value of each class. First, the median test value was computed for each class. These values were then ordered from least to greatest value. Next, the mean of the medians for all successive pairs of classes was used as a threshold to discriminate each class *i* from *i* + 1. For illustrative purposes, a binary and multi-class example is provided in Fig. 1.

Samples were classified based on where the test value fell in the list of means of ordered medians. Suppose there are *n* > 2 classes (*c*_*i*_ for *i* = 1, …, *n*), which result in *n*-1 threshold values (*t*_*i*_ for *i* = 1, …, *n* − 1). Further, suppose the test value of a sample *k* is denoted by *v*_*k*_. Then, typically, a sample *k* is classified as *c*_1_ if *v*_*k*_ < *t*_1_, *c*_*i*_ if *t*_*i* − 1_ ≤ *v*_*k*_ < *t*_*i*_ for *i* = 2, …, *n* − 1, and *c*_*n*_ if *v*_*k*_ ≥ *t*_*n* − 1_. However, the inequality signs can reasonably vary between > and ≥ and between < and ≤ for any of the inequalities. There are a total of 2^(*n* – 1)^ ways of classifying a group of samples. We consider all combinations and retain the classifier with highest classification accuracy.

The combination of a classifier equation, threshold values, and threshold behaviors are collectively known as a classifier. Classifiers that demonstrate the highest classification accuracy and lowest model complexity will survive the evolutionary process and be considered as a best classifier. The variables in the equation (i.e. set of features), which are gene-level RNA-Seq data in this study, can be inferred to be important biomarkers.

### Competing classification methods

The performance of the multi-class CES was evaluated against three competing feature selection and classification methods. Performance was assessed by measuring classification accuracy, the number of selected features, and stability of the selected feature sets across the 50 folds generated by 10 repeats of 5-fold cross validation (the details of 10 rep, 5-fold CV are described in a subsequent section). In what follows, we provide a general description of each method, state the R package used to carry out each analysis, and describe parameter settings. Briefly, we used the same default parameter settings for each dataset. For CES, the number of generations of evolution in each run was set to 1000 and the classifier solution base grid dimension was set to 36.

### Random forest

Random forest is a popular ensemble-learning classification algorithm that constructs a large number of decision trees and predicts the classes of observations by majority voting [10]. An importance score for each feature is also generated. Decision trees are binary classification trees where each node represents a feature in the dataset. These nodes as well as decision rules called ‘predicates’ are used to determine the classes of the observations, which are represented by the leaf nodes on the tree. The predicates are chosen by calculating information gain of each attribute, the amount of entropy reduced by discriminating based on that attribute. Less entropy means less variance in the resulting division of the data. Attributes with the most information gain are closest to the root of the tree. Over-fitting is prevented by pruning the tree after it has been built to find its most efficient and accurate form [4].

We utilized the *varSelRF* [11] to perform random forest classification in R. Default parameters were used for every dataset, following Fortino et al. [12]. Stopping criterion was equal to 1 times the standard deviation of the error. The number of variables randomly sampled at each split was equal to sqrt(p), where p is the number of features. The number of trees for the first forest was set to 5000. The number of trees for subsequent forests was set to 2000. The fraction of variables with low importance to exclude at each iteration was set to 0.2.

### Random k-nearest neighbor

K-nearest neighbor (KNN) algorithms use nonparametric classification rules based on training data that generalize well to the testing data [13]. Nonparametric algorithms are advantageous when the distribution is either unknown or hard to model, which is common in high-dimensional data. KNN also has the ability to impute values for missing data points, add flexibility and robustness, and simplify the preprocessing [14]. In this study, we performed random KNN (RKNN) [14], a generalization of KNN that uses an ensemble of base models, to perform random forest with feature selection for the high-dimensional datasets. Recursive backward elimination feature selection was performed using the default settings for all datasets. These settings included: *k* = 1 nearest neighbors, the number of KNN regressions was set to 500, the number of features drawn for each base KNN regression was set to $m=\sqrt{p}$, the proportion of the feature set to be kept in each elimination step was set to 0.5, and the minimum number of retained variables was set to 4.

### Support vector machines

Support vector machines (SVM) generate a maximal margin hyperplane that best separates a set of training data points [15]. If a linear hyperplane does not separate the data well, SVM algorithms can add additional variables to create nonlinear models or the data can be transformed into a higher dimension, where linear models can separate the training data well [16]. In R, SVM with feature selection was implemented differently than the other algorithms. There are only a few implementations of SVM with feature selection, and most are designed with built-in bootstrapping or cross validation. Furthermore, SVM methods that include feature selection normally report a rank for each feature, with the most important features ranked highest. The other methods in this study do not rank features but select a feature set. Thus, for comparison purposes, we used only the highest ranked features in the SVM and calculated metrics for the SVM method when the number of selected features was set to 4, 8, 16, 32, and 64. In order to carry out SVM with feature selection, we utilized the *OmicsmarkeR* [17] R package to rank features. Specifically, the function ‘svmrfeFeatureRankingForMultiClass’ was used with the following default parameters: the cost applied during model fitting was set to 1 and the percentage of features removed during each iteration was set to 10. The highest ranked 4, 8, 16, 32, and 64 features were used with the ‘svm’ function in the *e1701* [6] R package to train the models and make predictions. Default parameters were used for SVM, which is very robust to changes in parameters [18]. Specifically, the cost of constraints violation was set to 1 and a linear kernel was used.

### Datasets

Three real multi-class RNA-Seq datasets were used to carry out a comparative analysis of the classification algorithms. A summary of the number of samples, number of features, and number of classes for each dataset is provided in Table 1. The first dataset was generated from whole blood in rats to study the effects of amphetamine exposure and environmentally-induced hyperthermia (i.e. heat stroke). The dataset consisted of four classes (amphetamine hyperthermic, *n* = 20; amphetamine normothermic, *n* = 15; environmentally-induced hyperthermia, *n* = 22; and control, *n* = 16) with a total sample size of 73. RSEM (RNA-Seq by Expectation Maximization) counts were generated by Expression Analysis Inc. [EA; Durham, NC]. This dataset is available on the Gene Expression Omnibus repository [19] (accession numbers GSE64778 and GSE62368).

**Table 1:** The characteristics of the three datasets

Dataset		#Samples	#Features	#Classes
Rat blood	Full Dataset	73	12,549	4
	Immune-related Genes	73	227	4
Human cancer		304	19,955	6
Human lymphoblastoid		465	23,722	5

In addition, since previous research has shown that amphetamine exposure and EIH cause an innate immune system response that is detectable in circulating blood [20], the rat blood mRNA expression dataset was further analyzed for the purpose of identifying important immune-related biomarkers. Thus, the dataset was reduced to include only a subset of 227 genes that are known to be related to immune function. The list of genes was generated primarily from human mRNA expression at BioGPS [21] with consultation to mouse mRNA expression in particular types of leukocytes. In addition, we utilized the NCBI database to ensure that the genes were immune-related in humans and mice.

The second dataset was downloaded from The Cancer Genome Atlas. The dataset contained six classes of human cancer (lung squamus cell carcinoma, *n* = 52; liver hepatocellular carcinoma, *n* = 50; testicular germ cell carcinoma, *n* = 51; esophageal carcinoma, *n* = 51; breast invasive carcinoma, *n* = 49; and thyroid carcinoma, *n* = 51) with a total sample size of 304. RSEM raw counts for the gene-annotated, level 3 data were used. In this study, a subset of ~50 samples was randomly selected for each cancer type. Thus, samples for each subtype were not selected based on homogeneous markers. Here, the goal of classification is to discriminate tissue-of-origin.

The third RNA-Seq dataset of genome data from lymphoblastoid cell lines was obtained from the Genetic European Variation in Health and Disease (GEUVADIS) sequencing project [22]. The dataset contained cell lines from 462 individuals sampled from five different European populations (CEPH, *n* = 94; Finns, *n* = 95; British, *n* = 94; Toscani, *n* = 93; and Yoruba, *n* = 89) for a total sample size of 465. The FPKM (fragments per kilobase million) values from this study were uploaded into R (https://r-project.org) using the *geuvPack* package [23].

### Data preprocessing

The RSEM values generated from the rat blood and human cancer studies were rounded to the nearest whole number. The rat blood and human cancer datasets were transformed using variance stabilizing transformation [24] in the *DESeq2* R package [25]. The FPKM values in the human lymphoblastoid dataset were transformed using log2(FPKM + 1). Features for which there were no samples with a raw count value greater than or equal to five were removed from the rat blood and human cancer datasets and were not included in the analysis. In addition, expert knowledge was given to CES by supplying it with top the 100 features with largest F-statistic (and *p*-value < 0.05).

### Assessing classification accuracy

In the binary CES, accuracy for each classifier was calculated using a balanced accuracy formula [1]:

$$ A=\frac{TP/\left( TP+ FN\right)+ TN/\left( TN+ FP\right)}{2}, $$

where TP is the number of true positives, FN is number of false negatives, TN is the number of true negatives, and FP is the number of false positives. A true positive (negative) was defined as the correct prediction of a sample belonging to class one (zero). A false positive (negative) was defined as an incorrect prediction of a sample that actually belongs to class zero (one). In the multi-class setting, a simpler accuracy formula was used:

$$ A=\frac{Correct\; predictions}{Incorrect\; predictions+ Correct\; predictions}. $$

### Assessing stability of the selected feature set

Algorithms that perform feature selection can be evaluated and compared based on how consistently they identify important features. An inconsistent algorithm will return different sets of features when run on the same data or on permutations of the data. This could happen when many of the features are irrelevant to the response, but the selected features do a reasonable job of predicting class membership by chance alone. Reproducibility of feature selection methods is desirable [17]. Thus, measuring the stability of a feature selection algorithm is important in evaluating the performance of a classifier.

The stability of the selected feature set was assessed using an adaptation of the Tanimoto distance between two sets of features, s and s', as described in [26]:

$$ {S}_s\left( s, s\hbox{'}\right)\kern0.5em =\kern0.5em 1-\kern0.5em \frac{\left| s\right|\kern0.5em +\kern0.5em \left| s\hbox{'}\right|\kern0.5em -\kern0.5em 2\left| s\cap s\hbox{'}\right|}{\left| s\right|\kern0.5em +\kern0.5em \left| s\hbox{'}\right|\kern0.5em -\kern0.5em \left| s\cap s\hbox{'}\right|} $$

,where |s| and |s’| are the number of elements in sets s and s’, respectively. A Tanimoto value of zero indicates that the two sets share no common features; a Tanimoto value of one indicates that the two sets share all features.

### 10 rep, 5-fold cross validation

To compare the four classification methods, a 10 repetition, 5-fold cross validation was performed for each algorithm on each of the three datasets, not including the immune-filtered rat blood mRNA expression data. The immune-filtered dataset was analyzed using a single rep of 5-fold CV. The ‘*caret*’ [27] R package was used to divide the data into testing and training sets. In each fold, 80% of the data was used in the training set, while 20% of the data was reserved for testing set.

Classification accuracy and Tanimoto distance are reported as the average across all 50 feature sets. For CES, in each fold of the CV, accuracy was computed for all classifiers from the highest level of the Pareto tournament; we retained the classifier with highest accuracy for our reported averages.

### Final CES runs

Finally, in order to summarize the performance of CES, CES was run 10 times on each dataset, using a different random seed with each run. All selected features were retained for subsequent analysis.

## Results

**Fig. 2:**
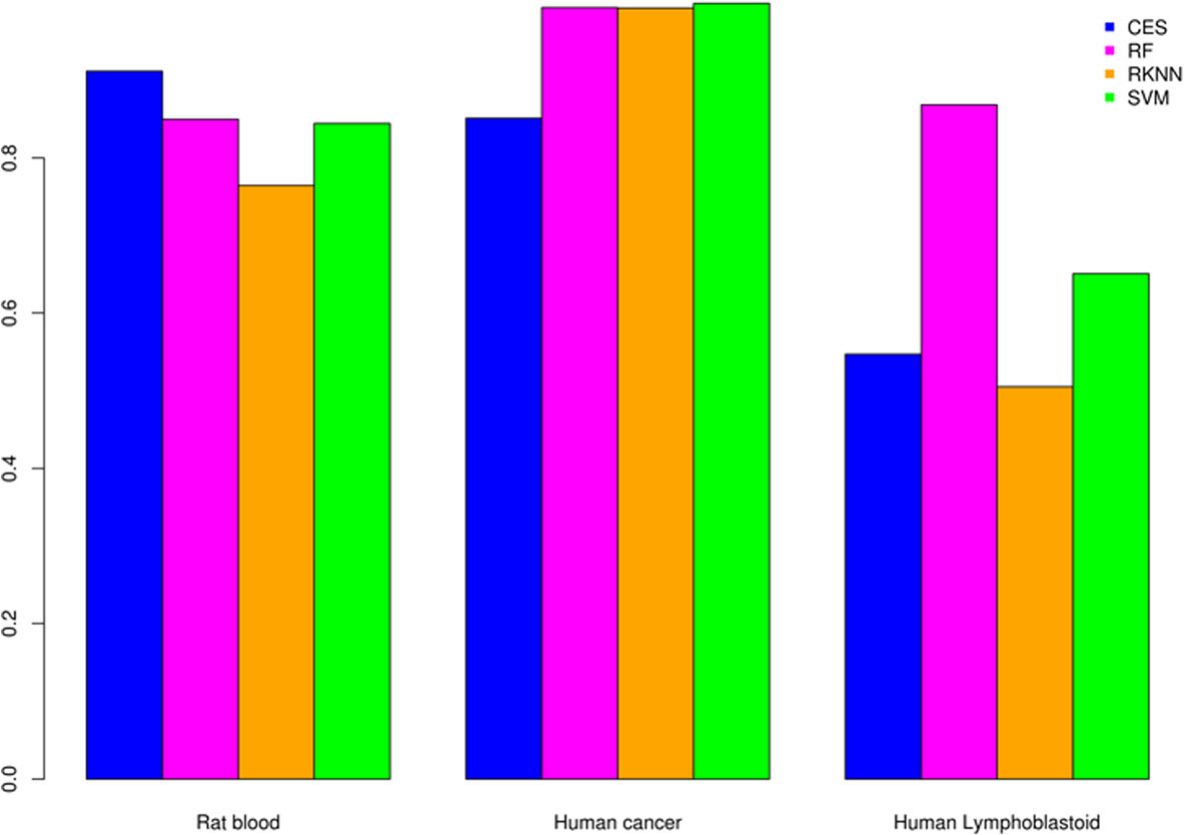
A summary of classification accuracy for each dataset.. Description of data: Classification accuracy was computed for each of the 50 testing datasets resulting from 10 Rep, 5-fold cross validation. We report the average across all evaluation datasets. Metrics are reported for CES, RF, RKNN, and the best performing SVM for all datasets

Results of the performance metrics for the rat blood, human cancer, and human lymphoblastoid datasets are presented in Tables 2, 3, and 4, respectively. In general, we find that the performance of the methods is data-dependent. By design, CES selects few features; the size of the feature set is considerably smaller than the competing methods. While CES attains highest accuracy for the rat blood dataset, it attains lowest accuracy for the human cancer dataset, and comparable accuracy, excluding RF, for the human lymphoblastoid data. A bar chart summarizing classification accuracy is presented in Fig. 2. Additionally, a comparative analysis of run-times is presented in Table 5.

**Table 2:** Rat blood mRNA expression 10 rep, 5-fold CV

Algorithm	Accuracy	Tanimoto Distance	Number of Selected Genes
CES	0.9113	0.2625	6
RF	0.8493	0.2406	453
RKNN	0.7645	0.0798	30
SVM	0.7034	0.2786	2
SVM	0.7858	0.2061	4
SVM	0.8031	0.2230	8
SVM	0.8284	0.3299	16
SVM	0.8438	0.3795	32
SVM	0.8428	0.4268	64

**Table 3:** Human cancer 10 rep, 5-fold CV

Algorithm	Accuracy	Tanimoto Distance	Number of Selected Genes
CES	0.8506	0.0957	8
RF	0.9933	0.2737	8188
RKNN	0.9927	0.9441	19381
SVM	0.8853	0.2785	4
SVM	0.9803	0.4454	8
SVM	0.9980	0.4739	16
SVM	0.9987	0.5215	32
SVM	1	0.5774	64

**Table 4:** Human lymphoblastoid 10 rep, 5-fold CV

Algorithm	Accuracy	Tanimoto Distance	Number of Selected Genes
CES	0.5468	0.2017	7
RF	0.8678	0.1795	4417
RKNN	0.5048	0.1539	50
SVM	0.4439	0.2382	4
SVM	0.5136	0.2921	8
SVM	0.5795	0.2678	16
SVM	0.6507	0.2846	32
SVM	0.7547	0.3441	64

**Table 5:** Algorithm run-time comparison for entire 10 rep, 5 fold CV

Algorithm	Run - time
CES	10 days
RF	25 min
RKNN	1 min
SVM	5 min

The run-time for CES in the cross validation was set to 1 day per fold. The Pareto tournament reaches a higher level when the run-time is extended. This corresponds to higher accuracies and simpler classifiers in each dataset. The 1 day run-time limited the Pareto level to 8 for the rat mRNA expression dataset, to level 6 for the human cancer dataset, and to level 5 for the human lymphoblastoid dataset. To explore possible increases in accuracy, simplicity, and Tanimoto distance, we performed a single rep, 5-fold cross validation for the cancer dataset with the run time set to 10 days. The results in Tables 6 and 7 show that allowing CES to run for longer periods of time will result in better classifiers.

**Table 6:** CES single rep, 5-fold CV performance with 1 day run - time

Pareto Level	Accuracy	Tanimoto Distance	Avg number of Selected Genes	Avg Number of Classifiers
6	0.7763	0.0873	10	5
5	0.8059	0.0871	8	14
4	0.8093	0.0938	8	32
3	0.8092	0.0871	9	64
2	0.7960	0.0941	7	121
1	0.8027	0.1336	6	216

**Table 7:** CES single rep, 5-fold CV performance with 10 day run - time

Pareto Level	Accuracy	Tanimoto Distance	Avg number of Selected Genes	Avg Number of Classifiers
9	0.9113	0.1080	6	5
8	0.9145	0.0863	7	9.4
7	0.9244	0.0713	7	23
6	0.9244	0.0742	7	46
5	0.9244	0.0605	9	92
4	0.9244	0.0846	9	179
3	0.9078	0.0697	10	351
2	0.9045	0.0911	9	672
1	0.8850	0.0800	8	1263

Classification accuracy for the immune-related rat blood dataset was 0.8210 for a single rep, 5-fold cross validation. Classification accuracy for the full rat blood dataset for the same 5 folds was 0.9457.

### Summary of features selected for the final CES run

The final CES run on each complete dataset was repeated ten times with different random seeds. The number of times each feature appeared in the final classifier is reported in Tables 8, 9, 10, and 11 for the rat blood dataset, the immune-related rat blood dataset, the human cancer dataset, and the human lymphoblastoid dataset, respectively. The listed features do not include genes that only appeared in one CES run.

**Table 8:** Rat blood selected genes

Gene	# of reps
Stip1	10
Enkur	9
Pea15a	8
Tpi1	3
Bst2	3
Gsg1	3
Hspa1b	3
Arf4	3
Dnaja1	2
MANF	2
Hsph1	2
Hsp90aa1	2
CREM	2

**Table 9:** Immune-related rat blood selected genes

Gene	# of reps
Cd96	10
Il12rb2	10
Ifitm1	9
Ifngr2	9
Il17ra	8
Cd44	7
Anxa2	5
Ccr3	5
Il1rap	4
Ifngr1	4
Cd300lb	4
Il7r	4
Il9r	4
Cd27	4
Il4ra	4
Ccl2	4
Il2rg	3
Ccl6	3
Il18bp	3
Cd84	3
Cd8a	3
Cxcl13	3
Il21r	3

**Table 10:** Human cancer selected genes

Gene	# of reps
DVWA	10
DUOXA1	10
TSHR	10
SLC23A2	8
VCP	8
SFTA3	8
NOX4	5
NKX2-1	4
HSD17B14	4
HPN	4
NKX2	4
POLR3A	3
DPYS	3
TRPS1	2
PTPRC	2
FGA	2
CDH3	2
HIST1H1T	2
UXT	2
NACAP1	2
PEBP1	2
ZNF706	2
CYP2C18	2
ASGR1	2
FGG	2

**Table 11:** Human lymphoblastoid selected genes

Gene	# of reps
ARHGEF18	10
RP11-108 M9.3	10
nckap5	7
IGLV2-5	5

### Detailed information for the rat blood selected genes

For each gene selected in the rat blood dataset, we retrieved human tissue and human or mouse cell type distribution information from BioGPS [21]. We also retrieved immune system related functions from the NCBI gene database [28] for the mouse and human, as these species have been studied more than the rat. This information is listed in Additional file 1: Table S1, which gives the details of the top 13 features most commonly selected by CES in the rat blood mRNA expression dataset (Table 8). Additional file 2: Table S2 gives details for the immune-related features most frequently selected by CES in the rat blood dataset (Table 9). For the genes listed in Additional file 1: Table S1 and Additional file 2: Table S2, the tissue/cell type distribution, immune function, and possible physiological importance relative to heatstroke and amphetamine toxicity are given.

## Discussion

A consistent advantage of CES is its ability to choose a very small number of features. Choosing a small number of features is important because returning a large number of selected features is indicative of model overfitting [29]. Additionally, a small number of selected features will also require less work for follow-up studies and further experimentation such as RT-PCR [29]. Finally, the few features identified by CES might increase the likelihood that said features would be important biomarkers. RF was inconsistent in the number of features it chose. On some folds RF chose a reasonable number of features (between ten and thirty), while most of the time, RF chose far too many features to be relevant for biomarker discovery. The number of features RKNN selected depended on the dataset. RKNN chose a reasonable number of features in the rat blood and human lymphoblastoid datasets, but in the human cancer dataset, RKNN eliminated almost no features.

Based on classification accuracy, CES performed better on the rat blood dataset than all other algorithms. This is especially impressive because this dataset had the smallest sample size. Datasets with smaller sample sizes are more difficult to accurately classify [30]. Classification accuracy for CES was 0.85 for the human cancer dataset. This performance is reasonably well; however, it should be noted that the other methods were near perfect. All methods, except RF, performed considerably worse on the human lymphoblastoid dataset. For this dataset, CES selected an average of 6 features and its classification accuracy was 0.55. Notably, its performance was better than SVM when we performed SVM with 4 and 8 of the top ranked features. Since SVM attained higher accuracy when the size of the feature set was set to 64, CES’s inherent ability to select a small feature set may limit its ability to achieve optimal classification.

Our findings for this multi-class study mimic those of similar studies i.e. no method completely outperformed the others [31]. According to the no free lunch theorem, no classification method is inherently superior for all classification tasks. In fact, many optimization methods may prove equal in terms of accuracy when the performance is averaged over all possible problems [32]. Considering the problem, the algorithm, and the data, certain algorithms will perform best on some datasets, while others perform better on a different dataset. Each method will lead researchers to different conclusions [17]. For example, Chai and Domeniconin [33] compared several feature selection methods for multi-class classification using several microarray datasets. SVM with recursive feature elimination performed best on datasets with a large number of features and classes, while correlation coefficients performed best on datasets with smaller dimensionality. The number of samples in each class as well as the amount of similarity between the classes [34] can affect the performance of a method. Therefore, it is unrealistic to expect any algorithm to be the best at all possible problems and datasets.

An advantage that CES has over other algorithms is that it can be used on any data type. CES can directly analyze data of any type, while other methods require data to be transformed so that the data is more normally distributed. In order to compare CES to competing methods, it was necessary to utilize variance stabilizing transformation [24] to transform the discrete counts generated by the RNA-Seq studies to a continuous scale. However, this step is not required for a CES as the methodology can process any data type.

The CES multi-classifier showed potential for identifying biomarkers associated with stress proteins, immune response, and toxicity in the full rat blood dataset (Table 8). Based on treatment means computed from DESeq2 normalized read counts, Triosephosphate Isomerase 1 (*TPI1)* and Germ Cell Associated 1 (Gsg1) were variably expressed in all four treatment groups, making them ideal discriminating features. Gsg1 is particularly intriguing since this gene is normally only found in the pineal and/or the testes in human (BioGPS).

In addition, gene ontology (GO) analysis of the features listed in Table 8 was performed using the Protein Analysis Through Evolutionary Relationships program (PANTHER, www.pantherdb.org). Protein folding (GO:0006457) was identified as a biological process that was significantly enriched. Four classic heat shock proteins were associated with this GO term. However, two very interesting genes coding for proteins regulating heat-shock protein activity in leukocytes (BioGPS) were not associated with protein folding but were listed in Table 8: tumor stress induced phosphoprotein 1 (*Stip1*) and bone marrow stromal cell antigen 2(*Bst2*). *Stip1* is expressed almost exclusively in B-Cell type leukocytes in human and probably rodents as well [21] and changes in its expression are of direct relevance to immune system status (BioGPS). Both genes may serve as biomarkers for leukocyte toxicity in blood.

Because amphetamine toxicity and hyperthermia are known to cause an immune response [35], we analyzed a subset of the rat blood dataset that comprised of immune-related genes. GO analysis was performed on features that were most commonly selected (Table 9). There was significant enrichment of several biological processes related to an innate immune response (e.g. lymphocyte chemotaxis (GO:0048247), chemokine-mediated signaling pathway (GO:0070098), inflammatory response (GO:0006954), granulocyte chemotaxis (GO:0071621), and lipopolysaccharide (GO:0032496)), which is likely due to increased numbers of monocytes [36] and possibly an upregulation of monocyte-specific genes in the AMPH and EIH groups. GO analysis also indicated that changes in the regulation of CD4-positive, alpha-beta T cell activation (GO:2000514) and positive regulation of T cell differentiation (GO:0045582) occurred. Finally, the CES selected the feature interferon induced transmembrane protein 1 (*Ifitm1*), which is also present primarily in T-Cells in humans and mast cells in mouse. The physiological implications of *Ifitm1* expression changes are unknown.

In the human cancer dataset, one would assume that many of the selected features may not be discriminant due to cancer but may be differentially expressed even in healthy subjects; we observed this for 17 of the 27 genes in Table 10. However, ten potentially cancer-dependent transcript classifiers were identified. Three of these transcripts (DUOXA1, POLR3A and NACAP) have virtually the same expression in all six tissues in healthy subjects (BioGPS). Expression of the solute carrier family 23 member 2 (SLC23A2) has been shown to be a colon and gastric cancer biomarker [37, 38]. RNA polymerase III subunit A (POLR3A) is involved with autoimmune disease scleroderma, which is a risk factor for cancer [39]. Three transcripts had very similar expression (VCP, TRSP1 and HIST1T) in all tissues in healthy subjects (BioGPS). Valocin containing protein (VCP) has been identified as a drug treatment target for cancer treatment [40]. Hisotone cluster H1 family member T (HIST1H1T) has not been previously identified but is a possible transcript upregulated in cancer and potential cancer biomarker.

Interestingly, NADPH oxidase 4 (NOX4) is normally only expressed in kidney (not one of the six cancerous tissues in the human cancer dataset) and is downregulated in various cancers but upregulated in lung cancer [41]. Two of the transcripts identified as features are predominantly expressed in leukocytes (BioGPS). However, zinc finger protein 706 (ZNF706) is also highly expressed in laryngeal cancer [42]. Protein tyrosine phosphatase receptor type C (PTPRC) codes for the protein tyrosine phosphatase CD45 (tumor suppressor) which has been shown to be present in T-cell acute lymphoblastic leukemia [43]. Hydroxysteroid 17-beta dehydrogenase 14 (HSD17B14) has been identified as a predictive biomarker for successful breast cancer treatment with tamoxifen [44] but its expression varies among the six tissues in the dataset.

Overall, the method developed here provides a good basis for the multi-class CES. However, we acknowledge some of its limitations. First, the accuracy of CES could be improved in the future by testing different combinations of parameters that control CES, with the number of generations and the size of the solution grid being most important. Additionally, consideration of a weighting factor in Pareto optimization to lend more (less) weight to classification accuracy (model complexity) might relax the constraint on model size, allowing for slightly larger feature sets, which may yield better accuracy for some datasets. Furthermore, it would be interesting to consider the effects of alternative multi-objective optimization strategies such as the Utilitarian approach in lieu of Pareto optimization [45]. The variables selected by SVM, RF, and RKNN could also be given to CES as expert knowledge to increase accuracy. Lastly, classification accuracy is an unbiased way to compare multi-class classification algorithms when class sizes are balanced [36], and is often used to assess multiclass classifiers [4, 46]. Since the datasets in this study are relatively balanced in regard to class distribution, the use of classification accuracy is fitting. However, in such cases where class size is imbalanced, metrics like F-score or macro-averaging accuracy would be better suited [47].

The main disadvantage of a CES is that it is computationally intensive. The reason is that it performs a coarse-grained genetic programming search and a fine-grained stochastic search of an infinite rugged fitness landscape [8]. The run-times in this study do not reflect a typical run-time for this algorithm, however. The run-times reported were for the entire 10 rep, 5-fold cross validation that was performed. Typically, an algorithm would be performed once on the full dataset. The CES can be run for as long as is desired. On a typical, modern computer, 15 h to one day of run-time is good enough. The cross validation was run in parallel on an Intel Xeon chip with 14 physical cores and 28 software cores. Five of the software cores were dedicated to the Pareto tournament for each fold, so 5 folds (i.e. a single rep), could be analyzed in parallel. Run-time for each fold of the cross validation was set to 24 h, meaning it would take 240 h, or about 10 days to complete the entire cross validation for a single dataset.

Using a large cluster computer would reduce the CES run - time, because the CES Pareto tournament is designed for parallel processing. More classifiers would be generated from different starting points in the rugged fitness landscape. These classifiers would be tested and mutated in parallel, and the resulting best classifiers would be used as starting points for CES runs in higher levels of the Pareto tournament. This ultimately leads to the identification of more accurate and less complex classifiers and more important biomarkers. The run-time of the multi-class CES algorithm could also be drastically reduced in the future by removing the attempt to optimize classification accuracy by testing all of the 2^(*n* – 1)^ combinations of threshold inequality signs for each classifier equation. Implementing future versions of the multi-class CES in this way would allow for comparable classification accuracies in less run - time since more classifiers would be generated in each level of the Pareto tournament.

## Conclusion

The multi-class extension of the CES can be used to identify important biomarkers in complex, multi-class datasets. The all-at-once multi-class classification approach allows for simpler implementation and interpretation of results compared to approaches that decompose the problem into multiple binary problems. This approach can be utilized in other algorithms to improve and streamline multi-class learning tasks.

## Additional files

Additional file 1: Table S1. Genes chosen by CES using all 12,549 genes expressed in rat blood. Description of data: A list of the most frequently selected genes in the rat blood dataset along with the tissues in which they are most highly expressed, their functions, and the reason why their expression would be increased. (PDF 69 kb)

Additional file 2: Table S2. Genes chosen by CES using 227 selected immune-related genes expressed in rat blood. Description of data: A list of the most frequently selected genes in the rat blood immune-related dataset along with the tissues in which they are most highly expressed, their function in relation to the immune system, and the reason why their expression would be increased. (TIF 4.17 kb)

## Abbreviations

CES: Computational evolution system
FLDA: Fisher linear discriminant analysis
FPKM: Fragments per kilobase of transcript per million mapped reads
HSP: Heat shock protein
KNN: K-nearest neighbor
RF: Random forest
RKNN: Random K-nearest neighbor
RSEM: RNA-Seq by expectation maximization
SDA: Symbolic discriminant analysis
SVM: Support vector machines
