# Supplementary material for: Multi-class computational evolution: development, benchmark evaluation and application to RNA-Seq biomarker discovery
Source: BioData Min. 2017 Apr 24;10:13. doi: 10.1186/s13040-017-0134-8 (PMC5404302; doi:10.1186/s13040-017-0134-8)
Supplement: Supplementary file 1 — Genes chosen by CES using all 12,549 genes expressed in rat blood. Description of data: A list of the most frequently selected genes in the rat blood dataset along with the tissues in which they are most highly expressed, their functions, and the reason why their expression would be increased. (PDF 69 kb) [file 13040_2017_134_MOESM1_ESM.pdf]

Genes most frequently chosen for classification using all 12549 genes expressed in rat b

NCBI Gene Symbol	# of reps selected	Tissue or Cell Type distribution[a]	Function[b]	Physiological import of increased or decreased expression of the transcript in blood
Stip1	10	High in leukocytes but present at appreciable levels in many tissues	Adaptor protein that coordinates the functions of HSP70 and HSP90 in protein folding; stimulates the ATPase activity of HSP70 and inhibits the ATPase activity of HSP90; regulates both the conformations and ATPase cycles of these [illegible]	Expression increases due to the extreme hyperthermia that can be produced by amphetamine exposure or EIH/ heat stroke

Enkur	9	No data available	This gene encodes a protein that interacts with calmodulin and several transient receptor potential canonical cation channel proteins. The encoded protein may function as an adaptor to localize signal transduction machinery to [illegible]	Expression increases may be due to amphetamine induced catecholamine release.

Pea15a	8	Expression restricted to neuronal tissues in astrocytes	The PEA15 gene in humans encodes a death effector domain-containing protein that functions as a negative regulator of apoptosis. The encoded protein is an endogenous substrate for protein kinase C. This protein is also overexpressed in type 2 diabetes mellitus, where it may contribute to insulin resistance in	Very modest 35% increases in expression due to hyperthermia or amphetamine. Not clear as to what cell type or exosome in blood expresses gene.

Tpi1	3	Significant expression in many different tissues and cell types	This gene encodes an enzyme, consisting of two identical proteins, which catalyzes the isomerization of glyceraldehydes 3-phosphate (G3P) and dihydroxy-acetone phosphate (DHAP) in glycolysis and gluconeogenesis. Mutations in this gene are associated with triosephosphat [illegible]	3-fold increases in expression from amphetamine exposure independent of hyperthermia. Activation likely due to catecholamine release.

Bst2	3	Highly expressed in leukocytes	The specific function of the protein encoded by the bone marrow stromal cell antigen 2 is undetermined; however, this protein may play a role in pre-B-cell growth and in rheumatoid arthritis.	5-fold expression increases due to the extreme hyperthermia that can be produced by amphetamine exposure or EIH/ heat stroke
Gsg1	3	Only present in pineal (extremely high) and testis	Targets testis-specific poly(A) polymerase to the endoplasmic reticulum through protein-protein interactions [27]	Increased 3-fold by amphetamine (no hyperthermia) 10-fold by hyperthermia and 20-fold when amphetamine produces hyperthermia. May be due to unknown cell type entering the circulating blood from the pineal or testes

Hspa1b	3	Expressed in many tissues	Heat shock protein 70 family member; stabilizes existing proteins against aggregation and mediates the folding of newly translated proteins in the cytosol and in	Dramatic (1000-fold) expression increases due to the extreme hyperthermia that can be produced by amphetamine exposure or EIH/ heat stroke
Arf4	3	Expressed in many tissues	Stimulates the ADP-ribosyltransferase activity of cholera toxin and play a role in vesicular trafficking and as activators of phospholipase D	Slight increases (25%) in expression by amphetamine. Significance unknown.
Dnaja1	2	Expressed in many tissues	Heat shock protein 70 family member. facilitates protein folding, trafficking, prevention of aggregation, and proteolytic	(20-fold) expression increases due to the extreme hyperthermia that can be produced by amphetamine exposure or EIH/ heat stroke

MANF	2	Expressed in many tissues but highest in leukocytes and liver not brain in human	Reduces ischemic brain injury and promotes behavioral recovery in rats.[27]; a secreted protein which reduces endoplasmic reticulum (ER) stress and has neurotrophic effects on dopaminergic	Expression increases likely due to amphetamine induced catecholamine release affecting leukocyte function.
Hsph1	2	High levels in brain tissues and leukocytes	Not much known but a non-Hodgkin lymphoma therapeutic target [29]	Dramatic (1000-fold) expression increases due to the extreme hyperthermia that can be produced by amphetamine exposure or EIH/ heat stroke

Hsp90aa1	2	Expressed in many tissues	Inducible heat-shock protein that functions as a homodimer. The encoded protein aids in the proper folding of specific target proteins by use of an ATPase activity that is modulated by co-	Dramatic (10-fold) expression increases due to the extreme hyperthermia that can be produced by amphetamine exposure or EIH/ heat stroke
CREM	2	Extremely high levels in the pineal with some in testes and adrenal	This classic transcription factor gene encodes a bZIP transcription factor that binds to the cAMP responsive element. It is an important component of cAMP-mediated signal transduction during the spermatogenetic cycle, as well as other [illegible]	Increased 3-fold by amphetamine (no hyperthermia) 10-fold by hyperthermia or when amphetamine produces hyperthermia. Like Gsg1, increases may be due to unknown cell type entering the circulating blood from the pineal or testes.

blood
