# Supplementary figures and images for: Multi-class computational evolution: development, benchmark evaluation and application to RNA-Seq biomarker discovery
Source: BioData Min. 2017 Apr 24;10:13. doi: 10.1186/s13040-017-0134-8 (PMC5404302; doi:10.1186/s13040-017-0134-8)

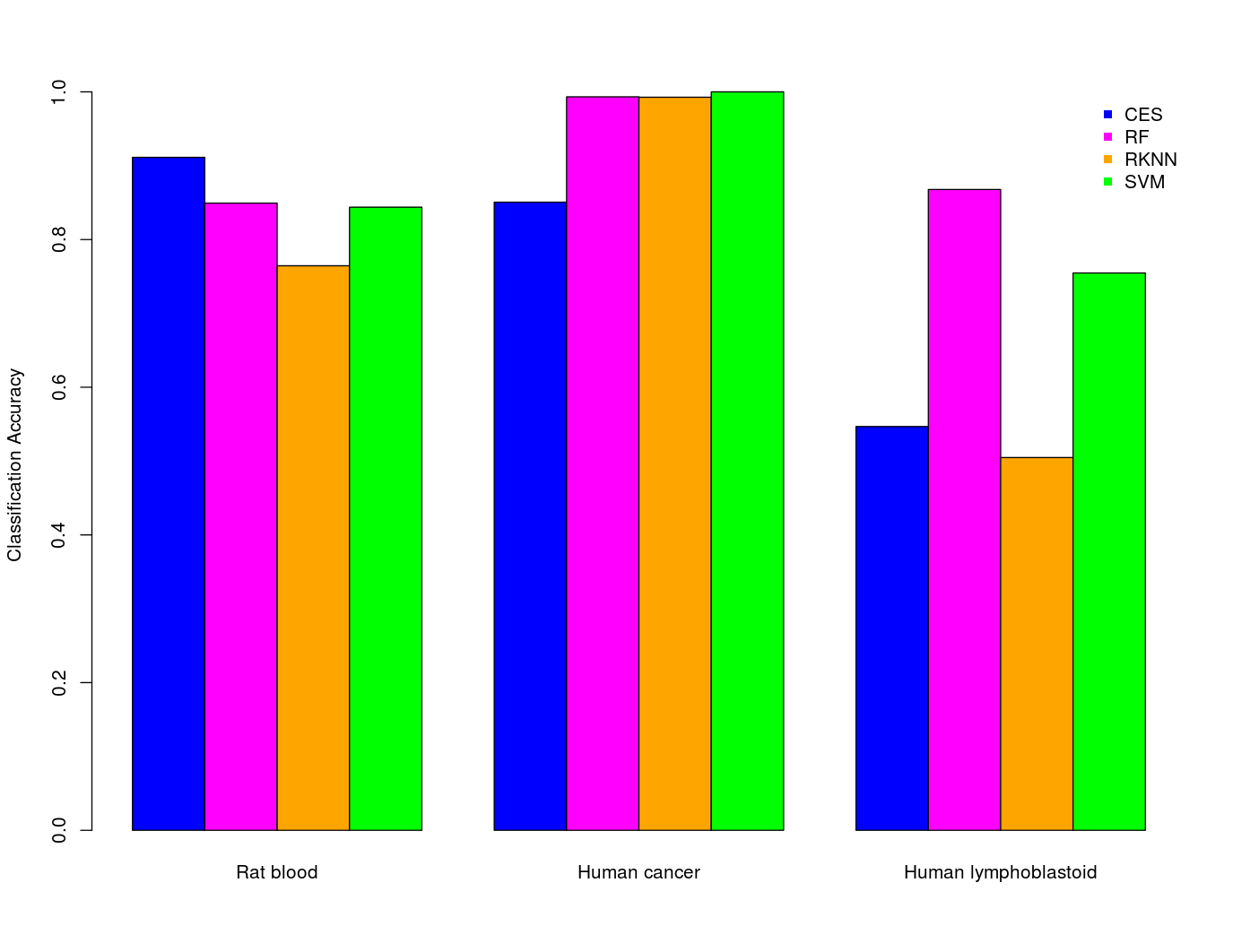

Supplement: Supplementary file 2 — Genes chosen by CES using 227 selected immune-related genes expressed in rat blood. Description of data: A list of the most frequently selected genes in the rat blood immune-related dataset along with the tissues in which they are most highly expressed, their function in relation to the immune system, and the reason why their expression would be increased. (TIF 4.17 kb) [file 13040_2017_134_MOESM2_ESM.tif]
